# Hepatitis B virus efficiently infects non-adherent hepatoma cells via human sodium taurocholate cotransporting polypeptide

**DOI:** 10.1038/srep17047

**Published:** 2015-11-23

**Authors:** Kaori Okuyama-Dobashi, Hirotake Kasai, Tomohisa Tanaka, Atsuya Yamashita, Jun Yasumoto, Wenjia Chen, Toru Okamoto, Shinya Maekawa, Koichi Watashi, Takaji Wakita, Akihide Ryo, Tetsuro Suzuki, Yoshiharu Matsuura, Nobuyuki Enomoto, Kohji Moriishi

**Affiliations:** 1 Department of Microbiology, Faculty of Medicine, University of Yamanashi, Japan; 2 Department of Molecular Virology, Institute for Microbial Diseases, Osaka University, Osaka 565-0871, Japan; 3 First Department of Internal Medicine, Faculty of Medicine, University of Yamanashi, Yamanashi 409-3898, Japan; 4 Department of Virology II, National Institute of Infectious Diseases, Toyama, Shinjuku-ku, Tokyo 162-8640, Japan; 5 Department of Molecular Biodefense Research, Yokohama City University Graduate School of Medicine Kanagawa 236-0004, Japan; 6 Department of Infectious Diseases, Hamamatsu University School of Medicine, Shizuoka, Japan

## Abstract

Sodium taurocholate cotransporting polypeptide (NTCP) has been reported as a functional receptor for hepatitis B virus (HBV) infection. However, HBV could not efficiently infect HepG2 cells expressing NTCP (NTCP-HepG2 cells) under adherent monolayer-cell conditions. In this study, NTCP was mainly detected in the basolateral membrane region, but not the apical site, of monolayer NTCP-HepG2 cells. We hypothesized that non-adherent cell conditions of infection would enhance HBV infectivity. Non-adherent NTCP-HepG2 cells were prepared by treatment with trypsin and EDTA, which did not degrade NTCP in the membrane fraction. HBV successfully infected NTCP-HepG2 cells at a viral dose 10 times lower in non-adherent phase than in adherent phase. Efficient infection of non-adherent NTCP-HepG2 cells with blood-borne or cell-culture-derived HBV was observed and was remarkably impaired in the presence of the myristoylated preS1 peptide. HBV could also efficiently infect HepaRG cells under non-adherent cell conditions. We screened several compounds using our culture system and identified proscillaridin A as a potent anti-HBV agent with an IC_50_ value of 7.2 nM. In conclusion, non-adherent host cell conditions of infection augmented HBV infectivity in an NTCP-dependent manner, thus providing a novel strategy to identify anti-HBV drugs and investigate the mechanism of HBV infection.

Hepatitis B virus (HBV) chronically infects approximately 3.4% of the world’s population and is a major factor for hepatocellular carcinoma following liver cirrhosis^1^. Interferon-alpha or nucleot(s)ide analogue inhibitors against the viral reverse transcriptase are currently approved for therapy for hepatitis B patients; however, these therapies are not necessarily effective for all such patients due to side effects and the emergence of escape mutant virus^2^. Thus, the development of new antiviral drugs that target several factors is still needed to prevent the liver diseases caused by HBV infection. Reliable and inexpensive cell culture systems and animal models of HBV infection are required in investigations of the underlying infection mechanism and pathogenesis of HBV. Although primary human hepatocytes (PHH), primary *Tupaia belangeri* hepatocytes (PTH), and the HepaRG cell line^3^ have been used as *ex vivo* HBV infection systems, these are typically employed under limited conditions, are expensive, and have difficulties maintaining stable susceptibility to HBV infection.

The HBV nucleocapsid is enveloped by a lipid bilayer enclosed within glycoproteins: the large (L), middle (M), and small (S) proteins of the HBV surface antigen (HBs)^4^. The L protein consists of preS1 and preS2 domains and the S protein, while the M protein consists of the preS2 domain and the S protein^4^. The S protein of the HBV virion has been shown initially, but weakly, to attach to heparan sulfate proteoglycans on hepatocytes^5^,^6^. Infection by HBV or hepatitis D virus (HDV) was previously reported to be neutralized by the antibody reacting to the preS1 region^7^ or by the myristoylated or acylated synthetic peptide composed of 47 N-terminal amino acids of the preS1 domain^8^,^9^,^10^, suggesting that the preS1 domain of the L protein is responsible for binding to the putative entry receptor(s). The sodium taurocholate cotransporting polypeptide (NTCP) was recently identified as a functional receptor for HBV and HDV because the myristoylated N-terminal region of the preS1 domain bound to NTCP and expression of NTCP rendered the HepG2 cell line susceptible to HBV infection^11^. The N-terminally myristoylated synthetic peptide corresponding to the region spanning from amino acid residue (aa) 2 to 48 of preS1 has been shown to interact with NTCP with high affinity^11^. The region spanning from aa 157 to 165 of NTCP was responsible for HBV infection and preS1 binding, while the region from aa 84 to 87 was for HBV infection but not preS1 binding^11^,^12^,^13^,^14^, suggesting that the region from aa 84 to 87 plays a role in a post-attachment step. Differences in these regions may determine host specificity for a member of the family Hepadnaviridae. Previous studies also suggested that the expression of NTCP provides HBV infectivity in the HepG2 cell line^11^,^15^,^16^,^17^. In the reported models, HBV could infect NTCP-expressing hepatoma cell lines under adherent monolayer-cell conditions^11^,^15^,^16^,^17^. However, NTCP-expressing HepG2 cells showed susceptibility to HBV infection compared with the parent cell line HepG2, but its infectivity was not high, which was indicated in the review process^11^. Schulze *et al.* reported that treatment with EGTA increased HBV infectivity in HepaRG cells^18^, suggesting that loosening of cell-cell junctions may promote HBV infectivity. Several reports suggest that NTCP is mainly expressed at the basolateral membrane of hepatocytes^19^,^20^,^21^. Thus, we hypothesized that the sufficient disruption of cell-cell junctions would expose NTCP to HBV virions in the medium, thereby promoting infectivity.

In the present study, we found lateral expression of NTCP in HepG2 cells transfected with the human *SLC10A1* gene (NTCP gene), investigated the effect of non-adherent cell conditions on HBV infection, and then established a novel cell culture system for NTCP-dependent HBV infection. We also examined the effects of several compounds on HBV infectivity by using our culture system.

## Results

### Establishment of HepG2 cell clones expressing NTCP

**Figure 1:**
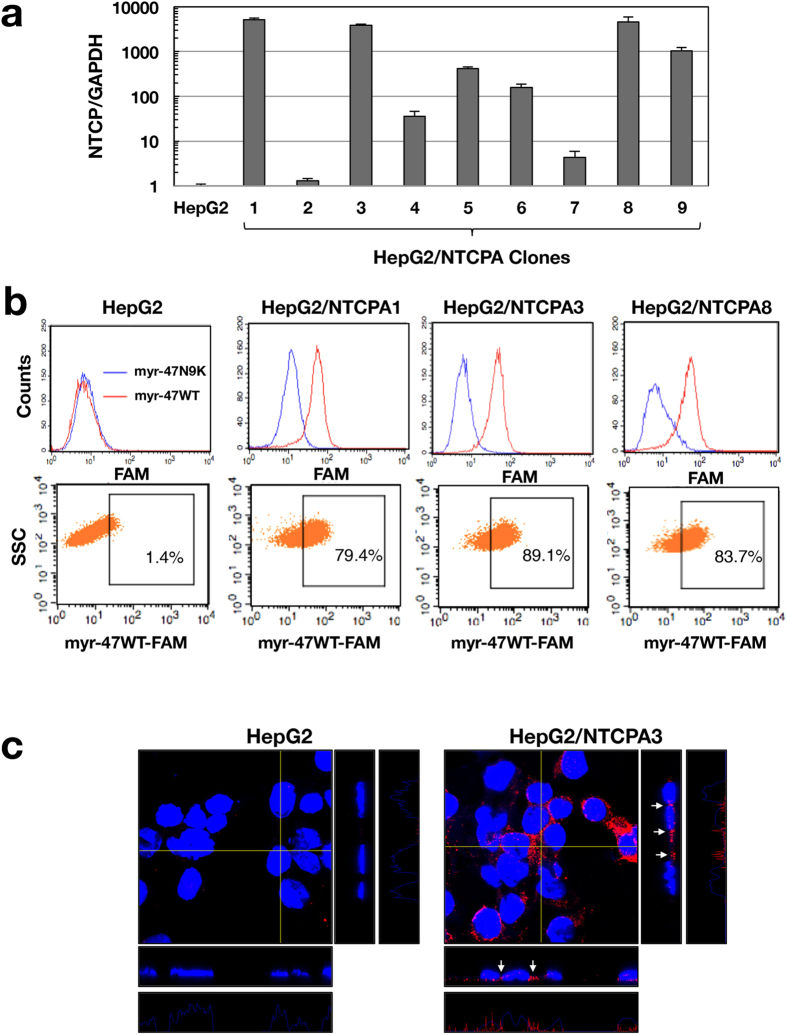
Establishment of a cell line expressing NTCP. (**a**) HepG2 cells were transfected with the plasmid encoding NTCP. The cells expressing NTCP were isolated as independent colonies and were then cloned by limited dilution. The amount of NTCP mRNA was evaluated by real-time qRT-PCR. HepG2/NTCPA1, 3, or 8 highly expressed NTCP. (**b**) Cell surface expression of NTCP was analyzed by FACS analysis using the lipopeptide myr-47WT. The lipopeptide myr-47N9K was used as a negative control. **(c)** HepG2 and HepG2/NTCPA3 cell lines were immunostained with the antibody to NTCP (red color) and counterstained with DAPI (blue color). These cells were observed under a confocal microscope. Orthogonal views (z-stacks) of each image are shown at the immediate right (yz planes) and lower (xz planes) of each main image. The z-stack step size was 0.5 μm. Arrows indicate the expression of NTCP in cell-cell contact regions. The fluorescent intensity profiles of NTCP in z-stacks are shown at the right end and bottom of each panel group.

The HepG2 cell line expressed NTCP at a low level (Fig. 1a) and did not exhibit any binding ability for the myristoylated preS1 peptide, myr-47WT, which specifically interacts with NTCP (Fig. 1b). HepG2 cells were transfected with the plasmid encoding NTCP and then incubated in medium containing puromycin. Viable cell clones were isolated as independent colonies and further cloned by limited dilution. Although we eventually established 9 clones expressing NTCP at various levels, HepG2/NTCPA1, 3 and 8 highly expressed NTCP (Fig. 1a). These cell clones were subjected to FACS analysis using the preS1 lipopeptide, myr-47WT, in order to evaluate the surface expression of NTCP. The HepG2/NTCPA3 cell line exhibited the greatest binding ability to myr-47WT among the three clones (Fig. 1b). Therefore, this cell line was subsequently used in experiments to evaluate HBV infectivity. NTCP was stained with anti-NTCP antibody and then observed by confocal microscopy. NTCP was localized in the lateral membrane and rather concentrated in the cell-cell contact region of HepG2/NTCPA3 cells under adherent monolayer-cell conditions (Fig. 1c), providing the hypothesis that non-adherent cell conditions of infection would enhance viral infectivity due to easy viral access to NTCP.

### Non-adherent cell conditions of infection augment HBV infectivity

**Figure 2:**
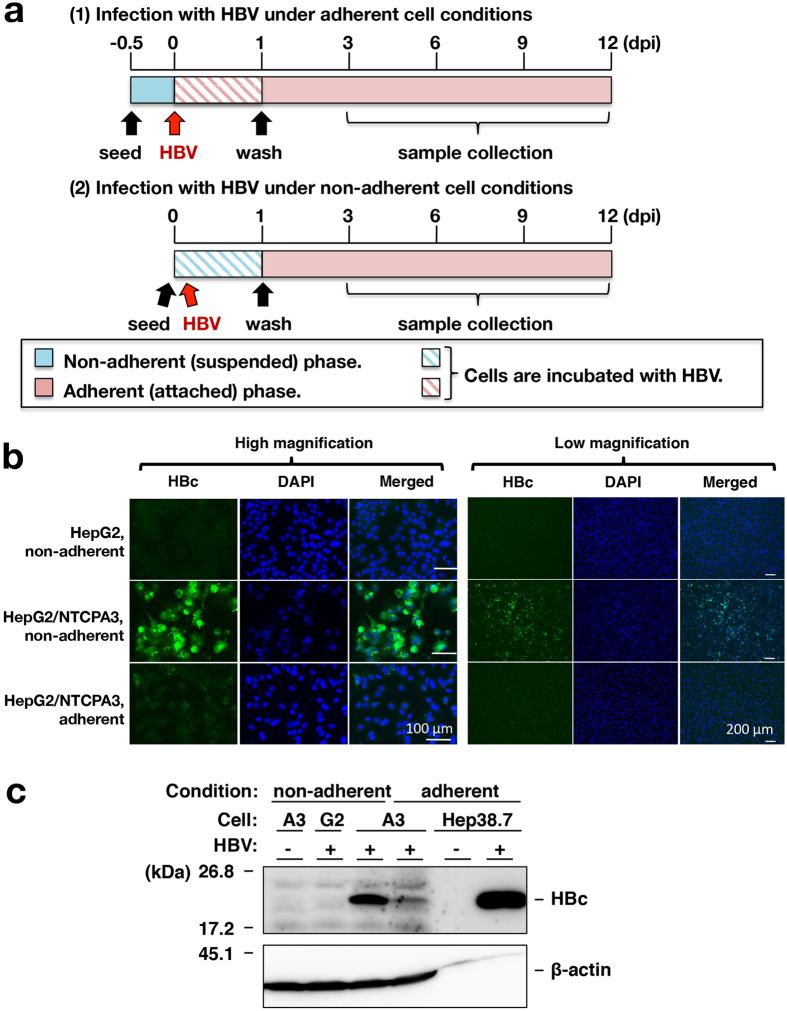
Enhancement of HBV infectivity by non-adherent cell conditions of infection. (**a**) Procedures for HBV infection of adherent (top) and non-adherent cells (bottom) are schematized. Non-adherent (suspended cells; blue bar) or adherent cells (attached cells; red bar) were infected with HBV by the method described in Materials and Methods. (**b**) HepG2 or HepG2/NTCPA3 cells were infected with HBV at 1,000 GEq/cell in non-adherent or adherent phase. Infected cells were fixed at 12 dpi and then stained with an antibody to HBc, and counterstained with DAPI. These cells were observed under a fluorescence microscope. (**c**) Non-adherent HepG2 cells, non-adherent HepG2/NTCPA3 cells, or adherent HepG2/NTCPA3 cells were infected with HBV at 1,000 GEq/cell and harvested at 12 dpi. The lysates of these cells and mock-infected non-adherent HepG2/NTCPA3 cells were subjected to Western blotting using an antibody to HBc. The lysate of Hep38.7-Tet cells maintained with (HBV: −) or without tetracycline (HBV: +) was applied together as a negative or positive control of molecular size. G2; HepG2 cells, A3; HepG2/NTCPA3 cells. Original blots are presented in Supplementary Figure 1.

**Figure 3:**
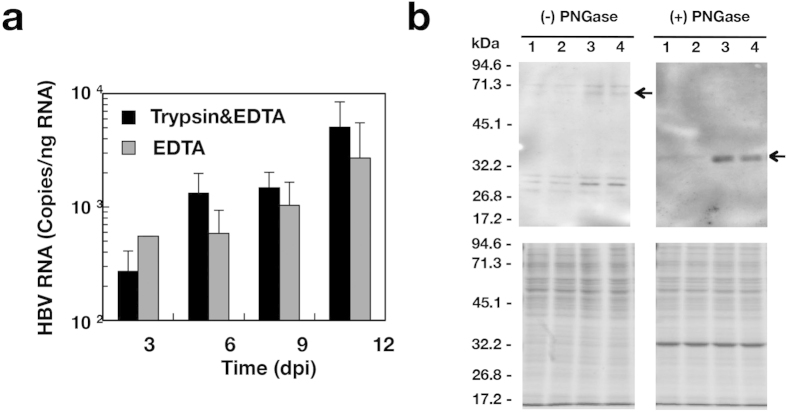
Effects of trypsin and EDTA on HBV infectivity and NTCP. (**a**) A cell suspension of the HepG2/NTCPA3 cell line was prepared with EDTA only or with both trypsin and EDTA. These suspended cells were infected with HBV at 1,000 GEq/cell. The resulting cells were harvested at the indicated time. The amount of intracellular HBV RNA was estimated by real-time qRT-PCR. Data were calculated from 4 independent experiments and are expressed as means ± SD. (**b**) HepG2 cells (lanes 1 and 2) or HepG2/NTCPA3 cells (lanes 3 and 4) were collected using a cell scraper (lanes 1 and 3) or by trypsin-EDTA treatment (lanes 2 and 4). The membrane fractions were prepared from the harvested cells and then incubated with (+) or without (−) PNGase for deglycosylation. These membrane proteins were subjected to Western blot analysis using an antibody to NTCP (upper panels) and CBB staining (lower panels).

We examined the effect of non-adherent conditions on HBV infectivity. The procedures of infection with HBV are described in the Materials and Methods section and summarized in Fig. 2a. HBV was added to adherent monolayer cells at 12 hours after seeding cells (Infection of adherent cells), while suspended cells were mixed with HBV and then incubated in each well of a plate (Infection of non-adherent cells). Cell suspension was prepared by trypsin-EDTA treatment, and HBV infected HepG2/NTCPA3 cells at 1,000 GEq/cell under adherent or non-adherent cell conditions. When HBV infected HepG2/NTCPA3 cells at 1,000 GEq/cell in adherent phase, HBc antigen was slightly detected in the cells by immunofluorescence assay (Fig. 2b, bottom panel). In contrast, HBc antigen was detected in most of the cells infected in non-adherent phase (Fig. 2b, middle panel). The cells infected in non-adherent phase was similar to that infected in adherent phase regarding intracellular localization of HBc antigen irrespectively of staining strength (Figs 2b and 6). As shown in Western blotting data of Fig. 2c, the amount of HBc antigen increased in the cells infected with HBV in non-adherent phase, compared with the cells infected in adherent phase (Fig. 2c), suggesting that trypsin-EDTA treatment enhances the susceptibility of HepG2/NTCPA3 cell line to HBV infection. To examine the effect of protease activity on HBV infectivity of HepG2/NTCPA3 cells, cell suspension was prepared by trypsin-EDTA treatment or by EDTA treatment. Trypsin-EDTA treatment was similar to EDTA treatment with regard to HBV infectivity (Fig. 3a). Next, we prepared the membrane fraction from the cells detached by scraping or trypsin-EDTA treatment in order to observe the effect of these treatments on NTCP. Each sample was applied to a lane at an equal amount of protein (Fig. 3b, lower panels). Degraded bands of deglycosylated NTCP were not found in the membrane fraction of HepG2/NTCPA3 cells treated with trypsin and EDTA (Fig. 3b, a right panel). Glycosylated NTCP was weakly reacted with the rabbit anti-NTCP polyclonal antibody but was not degraded by trypsin-EDTA treatment compared with the controls (Fig. 3b, a left panel). The data shown in Fig. 3 suggest that the non-adherent conditions of infection rather than proteolysis are critical for the enhancement of HBV infectivity. It would be difficult to prepare a suspension of the HepG2-derived cell lines by the treatment with EDTA only because the percentage of dead cells was increased during repeated pipetting due to sticky characteristics. Thus, host cell suspension was prepared by trypsin-EDTA treatment for non-adherent cell conditions of infection.

**Figure 4:**
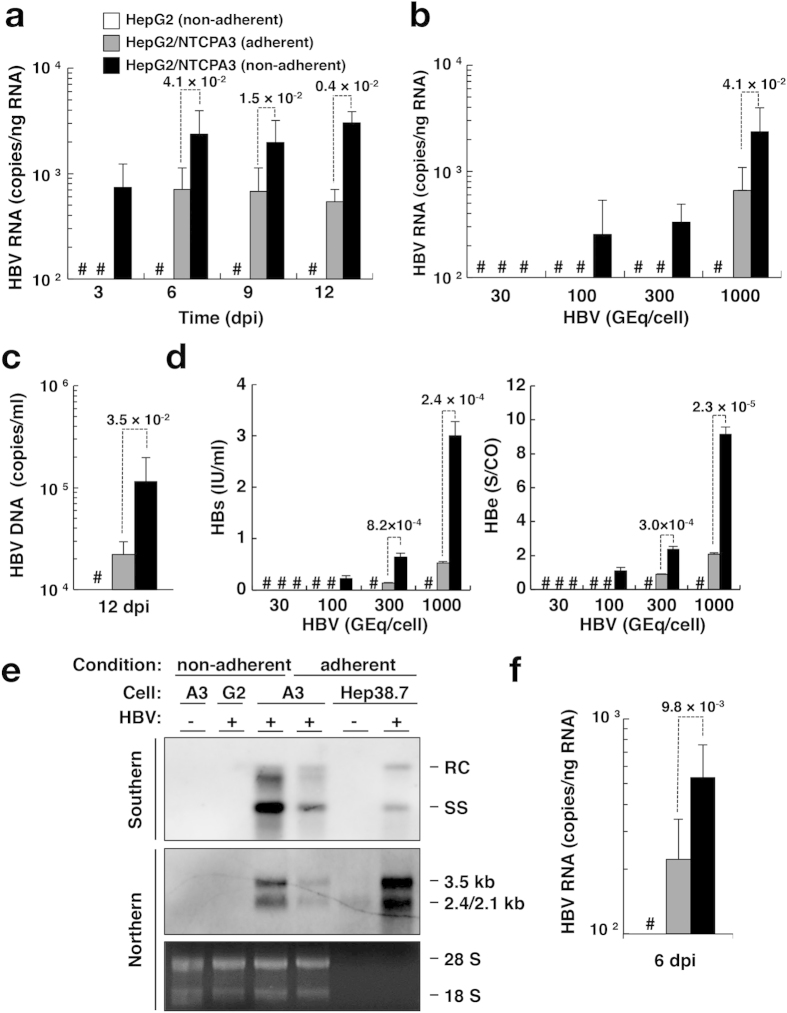
Effect of cell conditions on HBV infectivity. Non-adherent HepG2 cells (white bars), adherent HepG2/NTCPA3 cells (gray bars), or non-adherent HepG2/NTCPA3 cells (black bars) were infected with HBV. (**a**) Non-adherent or adherent cells were infected with HBV at 1,000 GEq/cell and harvested at the indicated dpi. The amount of intracellular HBV RNA was quantified by real-time qRT-PCR. (**b**) Non-adherent or adherent cells were infected with the indicated amount of HBV and harvested at 6 dpi. The amount of intracellular HBV RNA was quantified by real-time qRT-PCR. (**c**) Non-adherent or adherent cells were infected with HBV at 1,000 GEq/cell. Culture supernatants were collected at 12 dpi. The amount of supernatant HBV DNA was quantified by real-time qPCR. (**d**) Non-adherent or adherent cells were infected with the indicated amount of HBV. Culture supernatants were collected at 12 dpi. The amount of supernatant HBs (left half) and HBe (right half) antigens were quantified by a chemiluminescence immunoassay. (**e**) Non-adherent HepG2 cells, non-adherent HepG2/NTCPA3 cells, or adherent HepG2/NTCPA3 cells were infected with HBV at 1,000 GEq/cell (HBV: +). As a negative control, non-adherent HepG2/NTCPA3 cells were mock-infected (HBV: −). Hep38.7-Tet cells maintained with (HBV: −) or without tetracycline (HBV: +) were used as a negative or positive control of molecular size. Encapsidated HBV DNA was subjected to Southern blotting (top panel). Northern blotting of HBV RNAs was carried out by using total RNAs (middle panel). As a loading control for Northern blotting, 28 S and 18 S rRNAs stained with ethidium bromide are shown in a bottom panel. G2 and A3 indicate HepG2 and HepG2/NTCPA3, respectively. RC and SS indicate relaxed-circular and single-stranded, respectively, forms of HBV DNA. (**f**) Non-adherent or adherent cells were infected with blood-borne HBV at 1,000 GEq/cell. The resulting cells were harvested at 6 dpi. The amount of HBV RNA was quantified by real-time qRT-PCR. Data were calculated from 4 to 8 independent experiments and are expressed as means ± SD. The *p*-value obtained using Student’s *t*-test is indicated above the broken line of a bar pair. Number sign (#) indicates “a value was not detected”.

**Figure 5:**
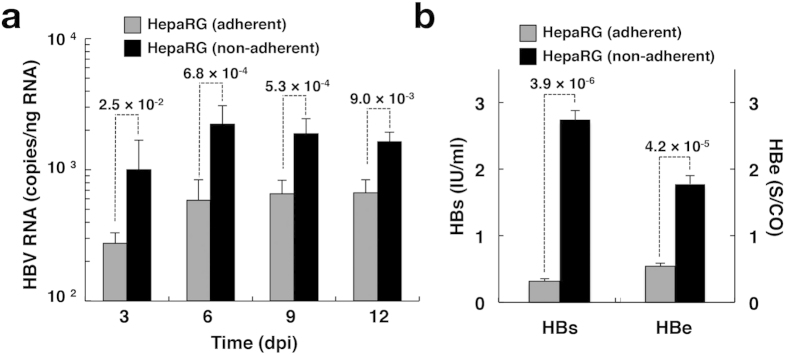
Infection with HBV in HepaRG cells. The differentiated HepaRG cells were infected with HBV at 1,000 GEq/cell in adherent (gray column) or non-adherent (black column) phase. (**a**) Infected cells were harvested at the indicated time. The amount of intracellular HBV RNA was quantified by real-time qRT-PCR. Data were calculated from 6 to 9 independent experiments. (**b**) Culture supernatants were collected at 12 dpi. The amount of supernatant HBs (left side) and HBe (right side) antigens were quantified by a chemiluminescence immunoassay. The *p*-value obtained using Student’s *t*-test is indicated above the broken line of a bar pair.

We examined the effect of non-adherent cell conditions on NTCP-dependent HBV infectivity and compared it to adherent cell conditions of infection. Intracellular HBV RNA was detected at 3 dpi and gradually increased until 12 dpi when HBV infected HepG2/NTCPA3 cells at 1,000 GEq/cell in non-adherent phase (Fig. 4a, black columns). On the other hand, intracellular HBV RNA of the group using adherent HepG2/NTCPA3 cells was detected from 6 dpi and was lower than that of the group using non-adherent HepG2/NTCPA3 cells (Fig. 4a, gray columns). Intracellular HBV RNA was detected when HBV infected HepG2/NTCPA3 cells at 100 or 300 GEq/cell in non-adherent phase (Fig. 4b, black columns). However, intracellular HBV RNA was not detected when HBV infected HepG2/NTCPA3 cells at 100 or 300 GEq/cell in adherent phase (Fig. 4b, gray columns). The amount of supernatant HBV DNA was significantly higher in the group using non-adherent HepG2/NTCPA3 cells than in the group using adherent HepG2/NTCPA3 cells (Fig. 4c). Productions of HBs and HBe antigens were also enhanced in the group using non-adherent HepG2/NTCPA3 cells compared with the group using adherent HepG2/NTCPA3 cells (Fig. 4d). HBs and HBe antigens were detected when HepG2/NTCPA3 cells were infected with HBV at 100, 300 or 1,000 GEq/cell in non-adherent phase (Fig. 4d, black columns). On the other hand, these antigens of the group using adherent HepG2/NTCPA3 cells (Fig. 4d, gray columns) were not detected or significantly lower than those of the group using non-adherent HepG2/NTCPA3 cells. To confirm the enhancement of HBV infectivity in the group using non-adherent HepG2/NTCPA3 cells, nucleocapsid-associated HBV DNA and intracellular HBV RNAs were analyzed by Southern and Northern blotting techniques, respectively (Fig. 4e). Relaxed-circular and single-stranded replicative DNA forms were produced in the group using non-adherent HepG2/NTCPA3 cells more highly than in the group using adherent HepG2/NTCPA3 cells (Fig. 4e, top panel). The data of Northern blotting also showed that HBV RNA was enhanced by using non-adherent HepG2/NTCPA3 cells (Fig. 4e, middle and bottom panels) in a similar way to the data shown in Fig. 4a,b. These findings suggest that HBV infects non-adherent HepG2/NTCPA3 cells more efficiently than the adherent cells. Blood-borne HBV could also efficiently infect non-adherent HepG2/NTCPA3 cells compared with the adherent cells (Fig. 4f). HBV did not infect the HepG2 cell line regardless of cell conditions or virus source (Fig. 4a,f). Additional four clones other than HepG2/NTCPA3 cells exhibited surface expression of NTCP and also showed higher HBV infectivity under non-adherent cell conditions than under adherent cell conditions, suggesting that enhancement of HBV infectivity in non-adherent phase is not limited in HepG2/NTCPA3 cells line (Supplementary Figure 2). We attempted to test whether or not the cell conditions of infection affect HBV infectivity to HepaRG cells. Although intracellular HBV RNA was gradually increased from 3 dpi to 6 dpi and maintained at a stable level between 6 and 12 dpi, the amount of HBV RNA was significantly higher at each dpi in the group using non-adherent HepaRG cells than in the group using adherent HepaRG cells (Fig. 5a). Similarly, the amounts of supernatant HBV antigens were significantly higher in the group using non-adherent HepaRG cells than in the group using adherent HepaRG cells (Fig. 5b). Hep38.7-Tet cells were maintained in the absence of tetracycline to induce HBV replication. When the HBV-replicating cells were detached by trypsin-EDTA treatment and re-seeded on the plate, the trypsin-EDTA treatment did not significantly affect the amount of intracellular HBV RNA (Supplementary Figure 3). These results suggest that non-adherent cell conditions of infection augment HBV infectivity but not the viral replication.

### Competitive inhibition of HBV infection with the preS1 peptide

**Figure 6:**
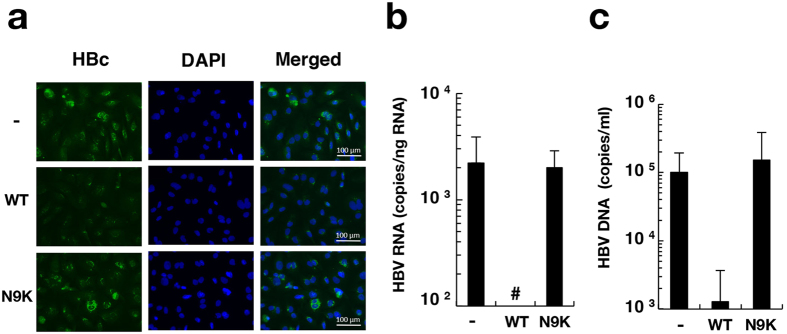
Inhibitory effect of preS1 lipopeptide on HBV infectivity. HepG2/NTCPA3 cells were infected with HBV at 1,000 GEq/cell under non-adherent conditions in the presence or absence of myr-47WT (WT) or myr-47N9K (N9K). (**a**) The infected cells were fixed at 12 dpi. The fixed cells were stained with an antibody to HBc and counterstained with DAPI. (**b**) The infected cells were harvested at 6 dpi. The amount of intracellular HBV RNA was quantified by real-time qRT-PCR. (**c**) The culture supernatants were harvested at 12 dpi. The amount of supernatant HBV DNA was quantified by real-time qPCR. Data were calculated from 4 to 6 independent experiments and are expressed as means ± SD. Number sign (#) indicates “a value was not detected”.

We examined the effect of preS1 peptide on HBV infectivity to non-adherent HepG2/NTCPA3 cells. The preS1 peptide myr-47WT or negative control myr-47N9K was incubated at 400 nM with cells for 1 h. Then, HBV was added to the cell culture supernatant and incubated for one day in the presence of a lipopeptide. The fresh medium lacking a lipopeptide was replaced with the culture medium at 1 dpi. The HBc antigen was detected at 12 dpi in untreated and myr-47N9K-treated cells, but not in myr-47WT-treated cells (Fig. 6a). Intracellular HBV RNA and supernatant HBV DNA were produced at a significantly higher level in untreated and myr-47N9K-treated cells than in myr-47WT-treated cells (Fig. 6b,c). These results suggest that HBV infects non-adherent NTCP-expressing cells via the preS1 region.

### Effects of taurocholic acid-uptake inhibitors on HBV infection

**Figure 7:**
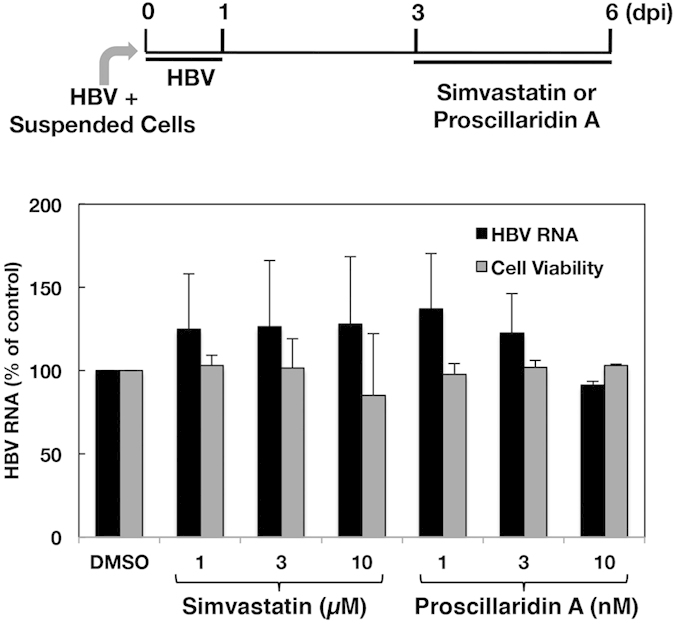
Effects of simvastatin and proscillaridin A on HBV replication. HepG2/NTCPA3 cells were infected with HBV at 1,000 GEq/Cell. Simvastatin or proscillaridin A was added to the culture supernatant at 3 dpi. The resulting cells were harvested at 6 dpi. The amount of intracellular HBV RNA was quantified by real-time qRT-PCR (black bars) and is presented as the percentage of the control value (DMSO). Cell viability was evaluated (gray bars). Data were calculated from 3 to 5 independent experiments and are expressed as means ± SD.

We screened the chemical compounds to identify anti-HBV compounds using our infection system with non-adherent cells. Simvastatin, fluvastatin, and digitalis-like compounds were previously shown to inhibit the uptake of taurocholic acid (TCA) via NTCP^10^,^11^. Furthermore, previous studies suggested that the inhibition of TCA uptake activity is associated with antiviral effects on HBV infection^22^,^23^,^24^. Thus, we investigated whether statins and digitalis-like compounds would impair HBV infection. Cells were treated with each compound from 1 h pre-infection to 1 dpi. Simvastatin inhibited HBV infection in a dose-dependent manner with an IC_50_ value of 5.2 ± 0.6 μM (Supplementary Figure 4a and Table 1), whereas fluvastatin did not inhibit it (Supplementary Figure 4b). Simvastatin and fluvastatin were not cytotoxic at 30 μM and 100 μM, respectively (Table 1). Proscillaridin A, a digitalis-like compound, also inhibited HBV infection and exhibited potent anti-HBV effects with an IC_50_ value of 7.2 ± 2.5 nM (Supplementary Figure 4c and Table 1), which is 2,000 times lower than the IC_50_ value for the inhibition of taurocholic acid uptake^25^. The CC_50_ value and selectivity index (SI) of proscillaridin A were calculated to be 495.9 ± 140.1 nM and 75.5 ± 26.7, respectively. Bufalin, convallatoxin, and digitoxin also inhibited HBV infection in a dose-dependent manner; however, these antiviral effects were weaker than those of proscillaridin A (Supplementary Figure 4d to f, and Table 1). On the other hand, digoxin did not inhibit HBV infection (Supplementary Figure 4g). A previous study that involved *in silico* analysis suggested that azelastine has putative binding activity to NTCP and anti-HBV activity^12^. In this study, azelastine exhibited moderate and significant inhibitory effects at 0.01 to 1 μM (Supplementary Figure 4h). The amount of supernatant HBe antigen was reduced in the presence of proscillaridin A (Supplementary Figure 4i), supporting anti-HBV effect of proscillaridin A on the basis of estimation of the viral RNA. To validate the utility of the infection system using non-adherent cells for drug screening, we investigated whether or not effect of proscillaridin A on HBV infectivity was varied by condition of infection. The anti-HBV effect of proscillaridin A in the group using non-adherent cells was similar to that in the group using adherent cells (Supplementary Figure 4j), suggesting that the non-adherent cell condition of infection is compared to the adherent cell condition of infection regarding drug screening for anti-HBV compound. When the HepG2/NTCPA3 cell line was treated with the compounds at 10 μM, compounds other than proscillaridin A did not impair the binding of myr-47WT (200 nM) to the cell surface of HepG2/NTCPA3 (Supplementary Figure 5). Proscillaridin A had a negligible effect on the binding of myr-47WT to the HepG2/NTCPA3 cell line; however, the inhibitory effect was not dependent on the concentration of proscillaridin A (Supplementary Figure 5e–g), suggesting that proscillaridin A nonspecifically and weakly influences the interaction between the preS1 peptide and HepG2/NTCPA3. Furthermore, the amount of HBV RNA was not decreased in the cells that were treated with simvastatin or proscillaridin A after infection (Fig. 7). The inhibitory function of proscillaridin A or simvastatin on HBV infection may be involved in a post-attachment step other than the viral replication or/and in indirect effect on binding affinity of NTCP to preS1.

## Discussion

In this study, NTCP was highly expressed in the lateral membrane and rather concentrated in the cell-cell contact region of HepG2/NTCPA3 cells (Fig. 1b). Thus, we hypothesized that the cell-cell junction disruption would enhance HBV infectivity due to easy access of HBV to NTCP. Our results in the present study indicate that HBV efficiently infects non-adherent HepG2/NTCPA3 cells compared with the adherent cells. HBV could also infect non-adherent HepaRG cells more efficiently than adherent cells (Fig. 5). Furthermore, HBV derived from a hepatitis B patient could efficiently infect non-adherent HepG2/NTCPA3 cells compared with the adherent cells (Fig. 4f). This was supported by the findings of Schulze *et al.* who found that the loosening of cell-cell junctions in hepatocytes increased HBV infectivity^18^. No significant difference was observed in HBV infectivity between cells treated with only EDTA and those treated with both trypsin and EDTA (Fig. 3a). Degraded bands of NTCP were not found in the cells treated with trypsin and EDTA (Fig. 3b). The data shown in Fig. 3 suggest that non-adherent conditions rather than proteolysis are responsible for the enhancement of HBV infectivity. Thus, our data in this study indicate that non-adherent cell conditions of infection augment HBV infectivity.

In the present study, we also tested the effects of several candidate compounds on HBV infection by using our cell culture system. Previous studies indicated that simvastatin, fluvastatin, and proscillaridin A interacted with NTCP at the bile acid binding pocket^25^,^26^. In addition, the administration of statin to hepatitis B patients was shown to reduce the risk of developing hepatocellular carcinoma^27^; however, the underlying mechanisms have not yet been clarified. In the present study, simvastatin, proscillaridin A, bufalin, convallatoxin, and digitoxin inhibited HBV infection in HepG2/NTCPA3 cells in a dose-dependent manner. Proscillaridin A in particular exhibited higher antiviral activity (Table 1). Proscillaridin A, which is one of the digitalis-like compounds, has been shown to inhibit NTCP-mediated taurocholic acid uptake with an IC_50_ value of 22 μM^25^, while it also showed anti-HBV activity with an IC_50_ value of 7.2 ± 2.5 nM (SI = 75.5 ± 26.7) (Table 1). As shown in Supplementary Figure 6 and Table 1, although bufalin has a similar chemical structure to proscillaridin A, its inhibitory effects on HBV infection are weaker than those of proscillaridin A. Proscillaridin A and convallatoxin possess L-rhamnose, whereas bufalin does not. Convallatoxin and digitoxin possess a different lactone ring from that of proscillaridin A (Supplementary Figure 6). These differences in chemical structure may affect the inhibitory effects of these compounds on HBV infection. Although simvastatin and fluvastatin were previously reported to inhibit NTCP-mediated taurocholic acid uptake at similar IC_50_ values^26^, HBV infection was inhibited by treatment with simvastatin, but not by treatment with fluvastatin, in the present study (Supplementary Figure 4a and b), suggesting that inhibition of the transporting activity of NTCP was not necessarily consistent with the inhibition of HBV infection. The results shown in Supplementary Figure 5 suggest that simvastatin and proscillaridin A inhibit HBV infection at a step other than the attachment of preS1 to NTCP. Treatment of the cells with simvastatin or proscillaridin A after infection did not exhibit an inhibitory effect on the viral replication (Fig. 7). Mouse NTCP could support the binding of preS1 peptide and the ability of taurocholate uptake, but not HBV and HDV infections^12^,^13^. These findings reported by two groups suggest that the region spanning from aa 84 to 87 in mouse NTCP is critical for HBV and HDV infections^12^,^13^. In fact, replacing the aa 84 to 87 region of mouse NTCP with the corresponding human region converted mouse NTCP into a functional receptor for HBV and HDV infection in HepG2 cells^12^,^13^, suggesting that NTCP is an entry factor responsible for the attachment and post-attachment steps of HBV and HDV infections. Thus, proscillaridin A or simvastatin may inhibit a post-attachment step before viral RNA replication. There is another possibility that proscillaridin A indirectly affects the affinity of NTCP to preS1 because of slight decrease in binding of preS1 to NTCP-expressing cells in the presence of these compounds (Supplementary Figure 5). Future study on the structure-activity relationship of digitalis-like compounds and/or statins may provide important information for the development of anti-HBV compounds and clarification of the role of NTCP in HBV infection.

In conclusion, these findings indicate that non-adherent cell conditions of infection augment HBV infectivity. Furthermore, our infection system using non-adherent cells could be employed for anti-HBV drug screening. Further screening of the chemical library by our infection system should contribute to the development of effective antiviral drugs to prevent HBV infection.

## Materials and Methods

### Plasmids

The gene encoding human NTCP was amplified from the human liver cDNA library for the Matchmaker yeast two hybrid (Clontech, Palo Alto, CA) and then introduced into pEF pGKpuro^28^ using the in-fusion HD cloning kit (Clontech). The resulting plasmid encoded human NTCP without an epitope tag.

### Cell lines

HepG2 cells were cultured in collagen-coated dishes and maintained in Dulbecco’s modified Eagle’s medium (DMEM; Sigma, St. Louis, MO, USA), supplemented with 10% fetal bovine serum (FBS; Sigma), 0.1 mM sodium pyruvate, and MEM non-essential amino acids (Gibco, Cambrex, MD, USA) at 37 °C in a 5% CO_2_ humidified incubator. HepG2 cells were transfected with the plasmid encoding human NTCP using the GenJet *In Vitro* Transfection Reagent (SignaGen, Gaithersburg, MD, USA). The transfected cells were cultured in medium containing 1 μg/ml puromycin. The viable cells were isolated as independent colonies and then further cloned by limited dilution. Finally, nine clones were established, as shown in Fig. 1a. HepaRG cells were purchased from Biopredic International (Rennes, France). HepaRG cells were cultured and differentiated according to the manufacturer’s protocol. HepaRG cells cultured at 2 weeks after differentiation were used for HBV infection, as described below.

### Preparation of HBV

HBV (genotype D) was prepared from the culture supernatant of the Hep38.7-Tet cell line^29^, which was derived from the HepAD38 cell line^30^. The Hep38.7-Tet cells were maintained in DMEM/F-12 (Sigma) supplemented with 10% FBS, 100 U/ml penicillin, 100 μg/ml streptomycin (Gibco), 18 μg/ml hydrocortisone (Sigma), 5 μg/ml insulin (Wako, Osaka, Japan), 400 μg/ml G418 (Nacalai Tesque, Kyoto, Japan), and 400 ng/ml tetracycline. The production of HBV was induced in the Hep38.7-Tet cell line by incubation in medium lacking tetracycline. The culture supernatant was harvested every 3 days between 7 and 30 days post-induction and was then filtrated through a 0.45-μm filter (Millipore Co., Billerica, MA, USA) to remove cell debris. The filtrated supernatants were concentrated using Vivaspin-100K (GE Healthcare Life Sciences, Uppsala, Sweden). HBV-positive sera were also used for HBV infection. The amount of HBV DNA was quantified by real-time PCR as described below.

### Infection with HBV under non-adherent or adherent cell conditions

In the case of the non-adherent cell conditions of infection, HepG2 cells, the derived cells, or differentiated HepaRG cells were harvested using 0.25% trypsin/ethylenediaminetetraacetic acid (EDTA) solution (Sigma). After washing once with 10% FBS-DMEM, the resulting cells were suspended in the primary hepatocyte maintenance medium (PMM) consisting of William’s E medium (Sigma) supplemented with 2 mM L-glutamine, 5 μg/ml transferrin (Sigma), 10 ng/ml recombinant human epidermal growth factor (PeproTech, Rocky Hill, NJ, USA), 3 μg/ml insulin, 18 μg/ml hydrocortisone, 40 ng/ml dexamethasone (Sigma), 5 ng/ml sodium selenite (Sigma), 2% DMSO (Wako), 100 U/ml penicillin, and 100 μg/ml streptomycin (Gibco). The suspended cells (1.25 × 10^5^ cells) were added to each well following the addition of virus, and were then immediately mixed well in 0.5 ml of PMM supplemented with 4% polyethylene glycol (PEG) 8000 (Sigma) and 2% FBS in each well of a 24-well collagen-coated plate. The resulting mixtures were incubated at 37 °C (0 days post-infection, dpi).

In the case of the adherent cell conditions of infection, the suspended cells were counted and seeded at 1.25 × 10^5^ cells/well in a 24-well collagen-coated plate. The resulting cells were incubated at 37 °C for 12 h in PMM supplemented with 10% FBS. The medium was replaced with fresh PMM containing 2% FBS, 4% PEG8000, and the indicated amounts of HBV. The cell plate was rocked well. The resulting cells were incubated at 37 °C (0 dpi).

The cells infected in non-adherent or adherent phase were washed three times with phosphate-buffered saline (PBS) at 1 dpi and incubated in fresh PMM supplemented with 10% FBS. The cells were re-seeded at 2 dpi at a density of 2 × 10^4^ cells/well (48-well collagen-coated plate) or 4 × 10^4^ cells/well (24-well collagen-coated plate) and maintained in PMM supplemented with 10% FBS. The cells and the culture supernatants were collected at each indicated dpi in order to evaluate the amounts of viral proteins, RNA, and DNA.

### Effects of the preS1 peptide or chemical compounds on HBV infection

Simvastatin, proscillaridin A, bufalin, convallatoxin, digitoxin, digoxin, and azelastine were purchased from Sigma. Fluvastatin was from Cayman Chemical Co. (Ann Arbor, MI, USA). The lipopeptide myr-47WT, which is an N-terminally myristoylated peptide consisting of aa 2 to 47 of the preS1 region of HBV, and myr-47N9K, in which Asn at the 9^th^ position is substituted with Lys, were synthesized by Scrum Co. (Tokyo, Japan)^11^. The lipopeptide myr-47N9K could not bind to human NTCP-expressing cells and was employed as a negative control^11^. The inhibition of infection was carried out using the following method: Suspended cells were prepared with 0.25% trypsin/EDTA solution and then washed three times with PMM. These cells (1 × 10^6^ cells/ml) were preincubated at room temperature for 1 h in PMM supplemented with a peptide at 400 nM or a compound at the indicated concentration, and were then infected with HBV at 1,000 GEq/cell in the presence of the preS1 peptide or compounds at the same concentration as in the preincubation. The resulting cells were washed three times with PBS at 1 dpi and incubated in fresh PMM supplemented with 10% FBS. Cells were then re-seeded at 2 dpi at a density of 2 × 10^4^ cells/well (48-well plate) or 4 × 10^4^ cells/well (24-well plate) and maintained in PMM supplemented with 10% FBS. The resulting cells and culture supernatants were collected at each indicated dpi in order to evaluate viral RNA and DNA.

The 50% inhibitory concentration (IC_50_) represents the concentration of a tested compound exhibiting half of the HBV RNA amount of a vehicle control. Cell viability was evaluated using the MTS assay system (CellTiter 96 AQueous One Solution Cell Proliferation Assay, Promega, Madison, WI, USA). The 50% cytotoxicity (CC_50_) represents the concentration of each compound exhibiting half of the cell-viability value of a vehicle control.

### Immunofluorescence microscopy

The cells grown on cover glass coated with 0.1 mg/ml collagen were washed twice with PBS and fixed with 4% paraformaldehyde (Wako). For HBc detection, these cells were permeabilized with 0.2% TritonX-100 (Wako) for 10 min, blocked with PBS containing 3% FBS, and then incubated at room temperature for 3 h with an appropriate concentration of a mouse monoclonal antibody to HBc (clone 7B2, a culture supernatant of the hybridoma)^23^. For NTCP detection, these cells were blocked with PBS containing 3% FBS, and then incubated at 4 °C overnight with 0.5 μg/ml rabbit anti-NTCP polyclonal antibody (anti-SLC10A1 antibody, Sigma). The treated cells were washed three times with PBS and reacted with 2 μg/ml AlexaFluor 488-conjugated goat anti-mouse IgG or or AlexaFluor 597-conjugated goat anti-rabbit IgG (Life Technologies) at room temperature for 1 h. The resulting cells were then washed three times with PBS, and were counterstained with DAPI (Sigma). Fluorescent images were obtained using a BIOREVO fluorescent microscope (Keyence Corporation, Osaka, Japan) or a FluoView FV1000 laser scanning confocal microscope (Olympus, Tokyo, Japan).

### Preparation and deglycosylation of membrane fractions

HepG2 cells and the derived cells cultured on 10-cm dishes were harvested using 0.25% trypsin/EDTA solution or a cell scraper. The cells were washed once with ice-cold PBS and then suspended in the homogenization buffer consisting of 5 mM Tris-HCl (pH7.5), 2 mM EDTA, 0.23 M sucrose, and a protease inhibitor cocktail (Roche, Indianapolis, IN). The resulting cells were homogenized with a Dounce homogenizer on ice, and centrifuged at 3,000 × g for 15 min at 4 °C. The supernatants were centrifuged again at 3,000 × g for 15 min at 4 °C. The resulting supernatants were further centrifuged at 100,000 × g for 30 min at 4 °C. The resulting precipitates as crude membrane fractions were solubilized in the lysis buffer consisting of 50 mM Tris-HCl (pH7.5), 150 mM NaCl, 1% Nonidet P-40, and a protease inhibitor cocktail. The protein concentrations of the lysates were determined using a DC protein assay kit (Bio-Rad). For deglycosylation, 10 μg of the membrane proteins were incubated with or without 1,000 U of PNGase F (New England Biolabs, Beverly, MA, USA) at 37 °C overnight. The resulting proteins were further mixed with SDS sample buffer, heated for 10 min at 60 °C, and analyzed by Western blotting and Coomassie Brilliant Blue (CBB) staining.

### Western blotting

For HBc antigen detection, HepG2 and HepG2/NTCP cells were harvested at 12 dpi and then lysed with the lysis buffer. The lysates were further mixed with SDS sample buffer and heated at 95 °C for 3 min. The lysates or membrane proteins were subjected to 12.5% polyacrylamide gel electrophoresis (SDS-PAGE). The lysate of Hep38.7-Tet cells maintained with or without 400 ng/ml tetracycline for 10 days was also applied to SDS-PAGE as a control of molecular size for the detection of HBc antigen. The proteins on the gels were electrically transferred to a PVDF membrane. The resulting membrane was blocked with Tris-buffered saline containing 0.05% Tween 20 and 5% skim milk (TBST) at room temperature for 1 h, and then incubated with TBST containing a mouse anti-HBc monoclonal antibody (1:500, Clone#7B2)^23^ or a rabbit anti-NTCP polyclonal antibody (150 ng/mL, Sigma) for 3 h or TBST containing a mouse anti-beta-actin monoclonal antibody (1:5,000; Sigma) at room temperature for 1 h. The membrane was washed three times with TBST lacking skim milk and then incubated with TBST containing 40 ng/ml horseradish peroxidase (HRP)-conjugated goat anti-mouse IgG antibody (Santa Cruz) or HRP-conjugated goat anti-rabbit IgG antibody (Santa Cruz) at room temperature for 1 h. After washing three times with TBST lacking skim milk, the proteins were visualized with the SuperSignal West Femto Maximum Sensitivity Substrate (Thermo Scientific, Rockford, IL, USA) and analyzed using the image analyzer LAS-4000 Mini (GE Healthcare, Tokyo, Japan).

### Estimation of viral DNA, RNA, and antigens

Total RNA was extracted using the RNeasy Mini Kit (Qiagen, Valencia, CA, USA), followed by DNase digestion using the TURBO DNA-free kit (Ambion, Austin, TX, USA). The resulting RNA preparation was reverse-transcribed using ReverTra Ace qPCR RT Master Mix (TOYOBO, Tokyo, Japan) according to the manufacturer’s protocol. Viral DNA was extracted from culture supernatants using the QIAamp DNA Mini Kit (Qiagen) according to the manufacturer’s protocol. The amounts of viral DNA and RNA were measured by real-time quantitative PCR (qPCR) and real-time quantitative reverse transcriptase (qRT)-PCR, respectively. The amounts of intracellular HBV RNA and supernatant HBV DNA were estimated according to the method described by Yan *et al.*^11^. The amounts of NTCP and GAPDH mRNAs were estimated by real-time qRT-PCR using the primer pairs 5′-CTCTCTTCTGCCTCAATGGAC-3′ and 5′-CAGTTGTGGCAGCTGTGTAG-3′, and 5′-GAAGGTCGGAGTCAACGGATT-3′ and 5′-TGATGACAAGCTTCCCGTTCTC-3′, respectively. Real-time PCR was carried out using the Fast SYBER Green Master Mix (Applied BioSystems) and StepOne Plus Real-Time PCR System (Applied BioSystems). The amounts of HBs and HBe antigens in culture supernatants were estimated by a chemiluminescent immunoassay (SRL Inc., Tokyo, Japan).

### Southern and Northern blotting techniques

HBV DNA probe was synthesized by using a DIG DNA labeling kit (Roche). The template DNA for the probe synthesis was gel-extracted from pUC-CAT digested by HindIII and EcoRI. For the synthesis of HBV RNA probe, HBV DNA flanked by the 5′ end of HindIII site and the 3′ end of KpnI site was amplified from pUC-CAT by PCR, and inserted into the KpnI and HindIII sites of pBluescriptII(SK-). The plasmid was linearized by HindIII. HBV RNA probe was synthesized from the linearized plasmid by using a DIG Northern Starter kit (Roche).

For the detection of encapsidated HBV DNA by Southern blotting, the cells infected with HBV at 1,000 GEq/cell were collected at 12 dpi, washed once with PBS and pelleted down by centrifugation. The cells were suspended and incubated in 0.5 ml of the lysis buffer consisting of 50 mM Tris-HCl (pH7.5), 1 mM EDTA and 1% Nonidet P-40 on ice for 15 min. The lysates were incubated with 10 U of DNase I and 0.02 mg/ml of RNase A at 37 °C for 2 hours, and then incubated with 200 μg/ml proteinase K in the presence of 100 mM NaCl, 16 mM EDTA and 1.5% SDS at 37 °C overnight. Nucleic acids were then extracted with equal amounts of phenol/chloroform, precipitated with 50% isopropanol in the presence of 300 mM sodium acetate and 80 μg glycogen, and rinsed with 70% ethanol. The precipitates were dissolved in TE buffer, loaded onto 1.2% agarose gel, and electrophoresed in TAE buffer. For the detection of HBV RNA by Northern blotting, total RNAs were extracted from the cells infected with HBV at 1,000 GEq/cell for 6 day. Total RNA (8 μg) was loaded in a lane on 1.2% agarose gel containing 18% formalin and electrophoresed in MOPS buffer. The bands of RNA in the gel were stained with ethidium bromide. The capillary transfer, hybridization, and detection were carried out in accordance with manufacture’s protocol. The signals were visualized with a CDP-star (Roche) and analyzed using the image analyzer LAS-4000 Mini.

### Flow cytometry using preS1 lipopeptides

HepG2 or HepG2/NTCPA3 cells were incubated at 1 × 10^6^ cells per 0.1 ml with 200 nM FAM-labeled myr-47WT or myr-47N9K for 30 min at room temperature, washed twice with PBS containing 2% FBS (2% FBS/PBS), and then incubated with 2 μg/ml propidium iodide (PI; BD Biosciences, San Jose, CA, USA) for 5 min. After washing twice with 2% FBS/PBS, cells were counted on a BD FACSCalibur (BD Biosciences) and data were analyzed using Cell Quest software (BD Biosciences).

### Competitive binding assay

HepG2/NTCPA3 cells were pre-incubated with simvastatin (10 μM), fluvastatin (10 μM), proscillaridin A (10 to 100 μM), or azelastine (10 μM) for 30 min at room temperature, washed twice with 2% FBS/PBS, and then incubated with 200 nM FAM-labeled myr-47WT or myr-47N9K in the presence of each compound (at each concentration). After washing twice with 2% FBS/PBS, the resulting cells were analyzed by FACS analysis.

### Statistical analysis

Data are shown as the mean ± standard deviation (SD). The significance of difference between two groups was determined using Student’s *t*-test. A significant difference was defined by a *p* value of less than 0.05. The significance of differences between more than two groups was determined in supplementary figure 4 using one-way ANOVA with Dunnett’s post hoc test (treated groups vs. the vehicle control group). When the test statistic of Dunnett’s test can reject the null hypothesis with alpha set at *p* < 0.05 (*) or *p* < 0.01 (**), the difference was represented as statistically significant. These analyses were carried out using StatMate III (ATMS, Tokyo, Japan).

### Ethics statement

Human sera were obtained from two hepatitis B patients who were followed up at Yamanashi University Hospital. The study protocol was approved by the Human Ethics Review Committee of Yamanashi University Hospital. This study followed the principles of the ethical guidelines of the Declaration of Helsinki. All participants provided written informed consent.

## Additional Information

**How to cite this article**: Okuyama-Dobashi, K. *et al.* Hepatitis B virus efficiently infects non-adherent hepatoma cells via human sodium taurocholate cotransporting polypeptide. *Sci. Rep.* **5**, 17047; doi: 10.1038/srep17047 (2015).

## Supplementary Material

Supplementary Figures

## Figures and Tables

**Table 1:** Anti-HBV activities of chemical compounds.

Compounds	IC_50_	CC_50_	Selectivity Index
Simvastatin	5.2 ± 0.6 μM	>30 μM	ND
Fluvastatin	>30 μM	>100 μM	ND
Proscillaridin A	7.2 ± 2.5 nM	495.9 ± 140.1 nM	75.5 ± 26.7
Bufalin	32.4 ± 11.3 nM	478.6 ± 64.7 nM	15.8 ± 4.3
Convallatoxin	10.7 ± 3.6 nM	155.7 ± 53.8 nM	15.7 ± 4.7
Digitoxin	93.3 ± 15.3 nM	1727.0 ± 384.2 nM	18.9 ± 3.4
Digoxin	>100 nM	1429.3 ± 276.6 nM	ND
Azelastine	>30 μM	>30 μM	ND

ND: not determined.
