# Supplementary material for: Hepatitis B virus efficiently infects non-adherent hepatoma cells via human sodium taurocholate cotransporting polypeptide
Source: Sci Rep. 2015 Nov 23;5:17047. doi: 10.1038/srep17047 (PMC4655410; doi:10.1038/srep17047)
Supplement: Supplementary Figures [file srep17047-s1.pdf]

Supplementary Information

Hepatitis B virus efficiently infects non-adherent hepatoma cells via human sodium taurocholate cotransporting polypeptide.

Kaori Okuyama-Dobashi[1], Hirotake Kasai[1*], Tomohisa Tanaka[1*], Atsuya Yamashita[1], Jun Yasumoto[1], Wenjia Chen[1], Toru Okamoto[2], Shinya Maekawa[3], Koichi Watashi[4], Takaji Wakita[4], Akihide Ryo[5], Tetsuro Suzuki[6], Yoshiharu Matsuura[2], Nobuyuki Enomoto[3], and Kohji Moriishi[1]

[1]Department of Microbiology, Faculty of Medicine, University of Yamanashi, Japan
[2]Department of Molecular Virology, Institute for Microbial Diseases, Osaka University, Osaka 565-0871, Japan
[3]First Department of Internal Medicine, Faculty of Medicine, University of Yamanashi, Yamanashi 409-3898, Japan
[4]Department of Virology II, National Institute of Infectious Diseases, Toyama, Shinjuku-ku, Tokyo 162-8640, Japan
[5]Department of Molecular Biodefense Research, Yokohama City University Graduate School of Medicine Kanagawa 236-0004, Japan
[6]Department of Infectious Diseases, Hamamatsu University School of Medicine, Shizuoka, Japan

Table contents

1. **Supplementary Figures (S1 - S6)**
2. **Supplementary reference**

Un-cropped blot images of FIG. 2c.

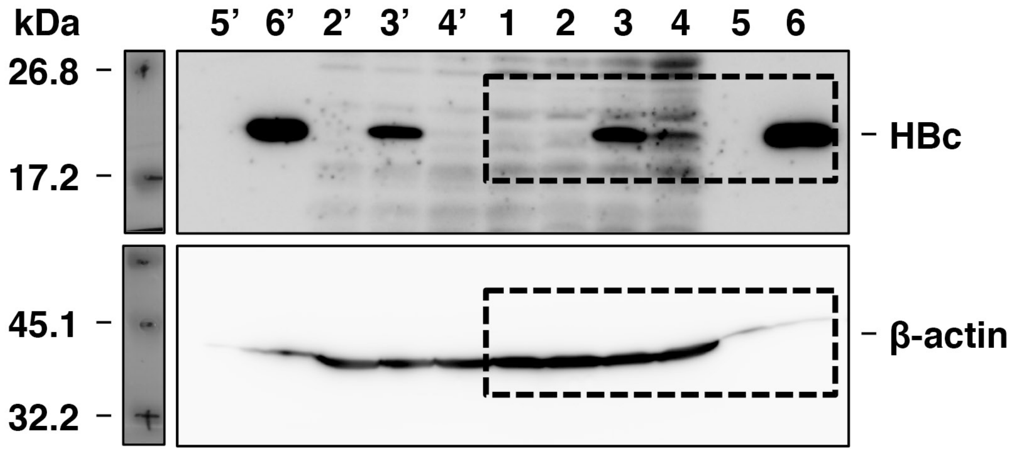

Supplementary Figure 1

Raw data of FIG. 2(c) were shown.

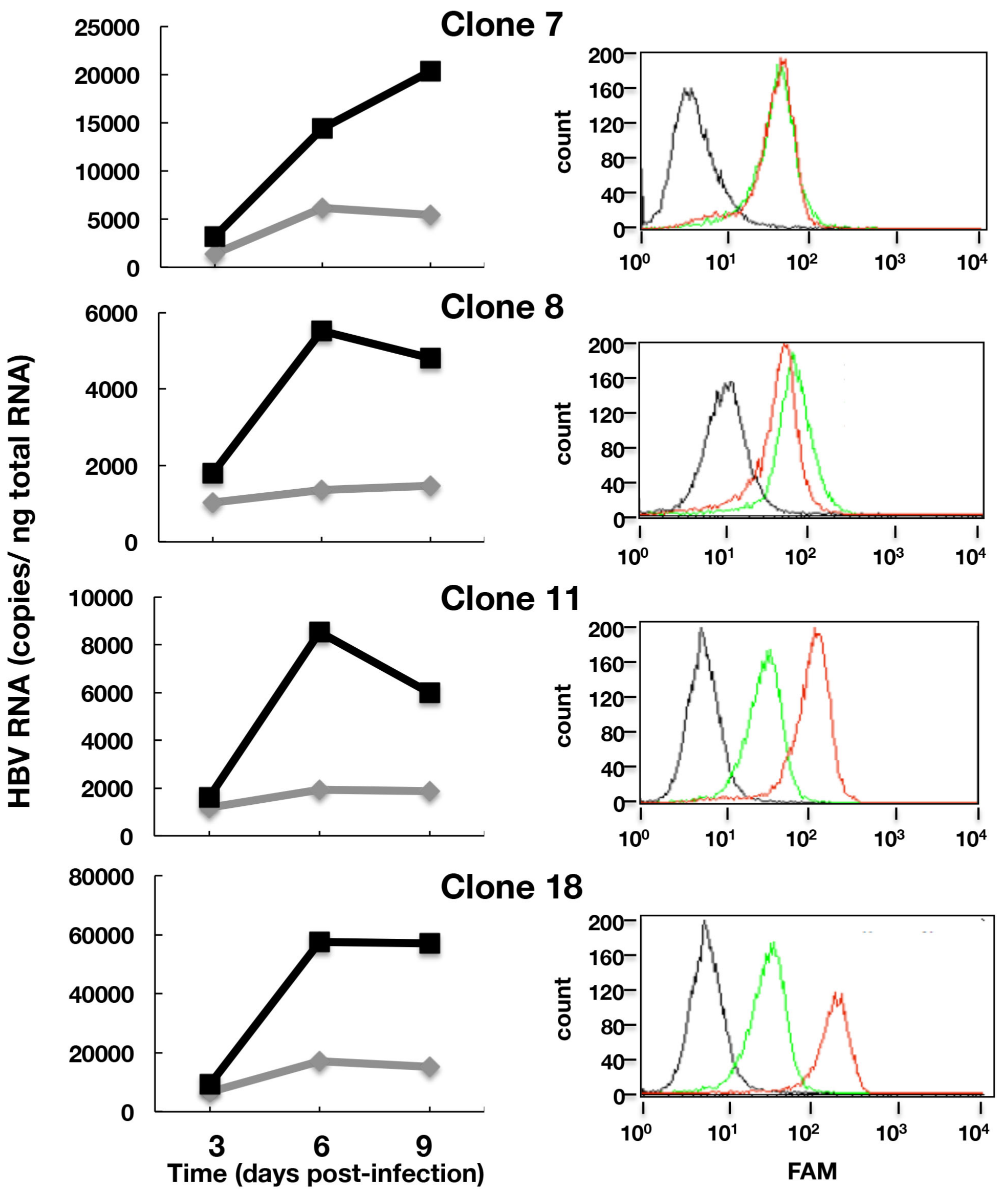

Supplementary Figure 2. Effect of cell condition on infections to other NTCP-expressing cell lines. **Left half:** Four independent HepG2 cell lines stably expressing NTCP (clone 7, 8, 11, and 18) were established from four colonies and then infected with HBV at 1,000 GEq/cell in non-adherent (black line) or adherent (gray line) phase. The amounts of intracellular HBV RNA were quantified by real-time qRT-PCR. **Right half:** Cell surface expressions of NTCP on NTCP-expressing HepG2 cell lines were analyzed by FACS analysis using the lipopeptide myr-47WT (red line). HepG2 cells (black line) and HepG2/NTCPA3 cells (green line) were used as negative and positive controls, respectively.

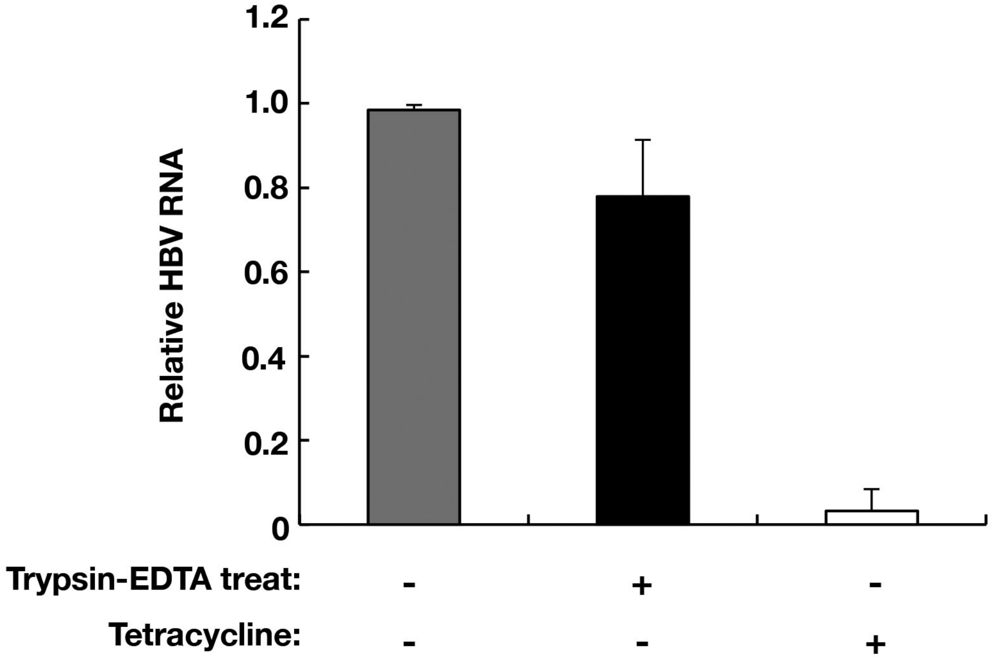

Supplementary Figure 3. Effect of trypsin/EDTA treatment on HBV replication. Hep38.7-Tet cells were seeded and then maintained in the absence of tetracycline (HBV-induced cells, day 0). One group of the HBV-induced cells was harvested at day 6 without trypsin-EDTA treatment (control group, gray bar). Another group of HBV-induced cells was detached at day 2 by trypsin-EDTA treatment and then reseeded immediately. The resulting cells were harvested at day 6 (black bar). Hep38.7-Tet cells were maintained with 400-ng/ml tetracycline (white bar). The amounts of intracellular HBV RNA were measured by real-time qRT-PCR. Data are calculated at the relative ratio to the value of control group.

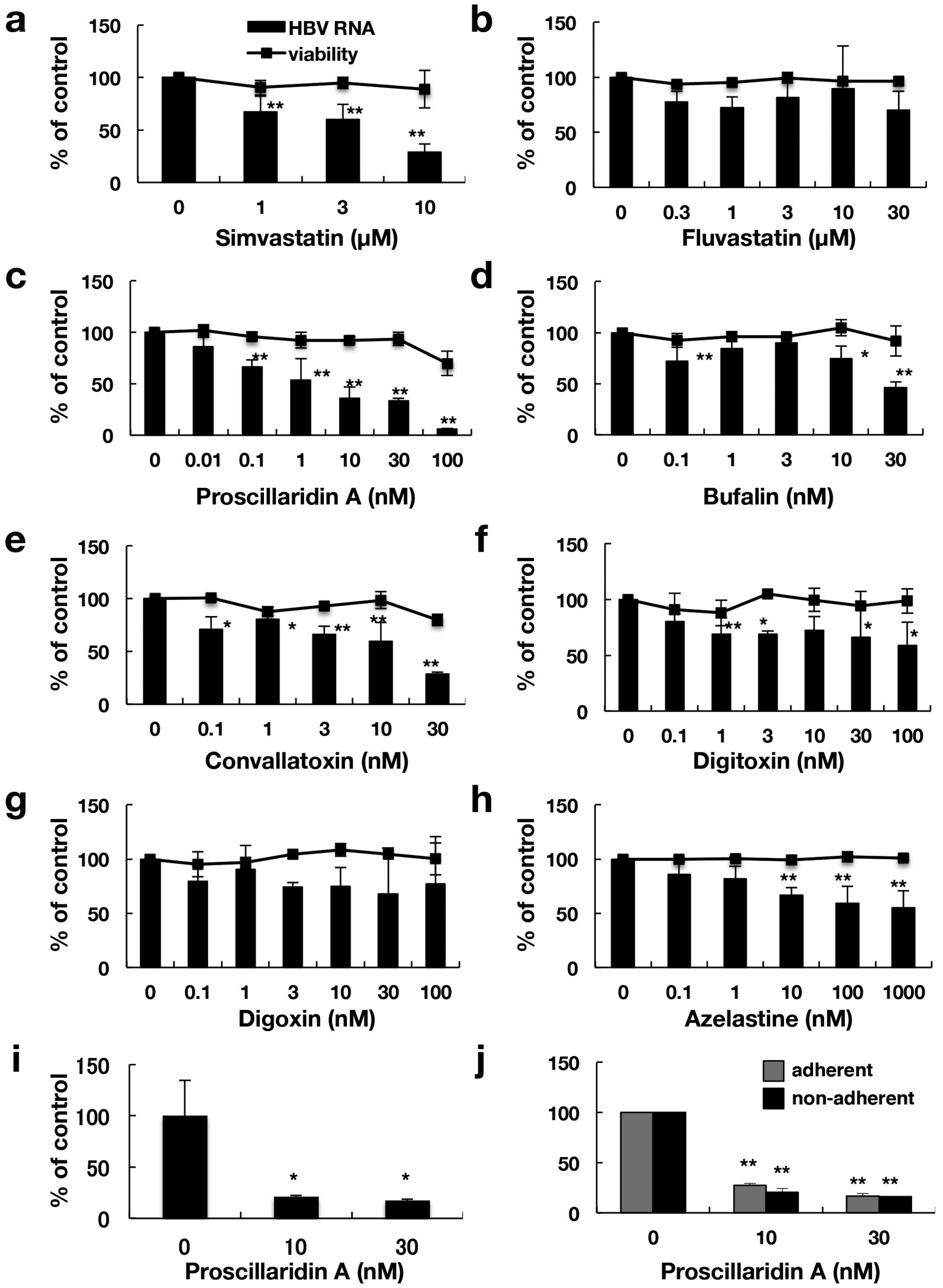

Supplementary Figure 4. Effects of chemical compounds on HBV infectivity.
A-to-H. HepG2/NTCPA3 cells were infected with HBV at 1,000 GEq/cell in non-adherent phase in the presence of the indicated concentration of simvastatin (a), fluvastatin (b), proscillaridin A (c), bufalin (d), convallatoxin (e), digitoxin (f), digoxin (g), or azelastine (h). The amount of intracellular HBV RNA was quantified by

real-time qRT-PCR in cells harvested at 6 dpi (black bars). Cell viability was measured by the MTS assay (black line with a triangle). **i.** Culture supernatants were collected at 12 dpi from HepG2/NTCPA3 cells that were infected with HBV at 1,000 GEq/cell in non-adherent phase in the presence of proscillaridin A. The amount of supernatant HBe antigen was quantified by a chemiluminescence immunoassay. **j.** HepG2/NTCPA3 cells were infected with HBV at 1,000 GEq/cell in adherent phase (gray columns) or non-adherent phase (black columns) in the presence of 0, 10 or 30 nM proscillaridin A. The amount of HBV RNA was quantified by real-time qRT-PCR. The difference of significances was estimated by using one-way ANOVA followed by Dunnett's post hoc test with alpha set at $p < 0.05$ (*) or $p < 0.01$ (**).

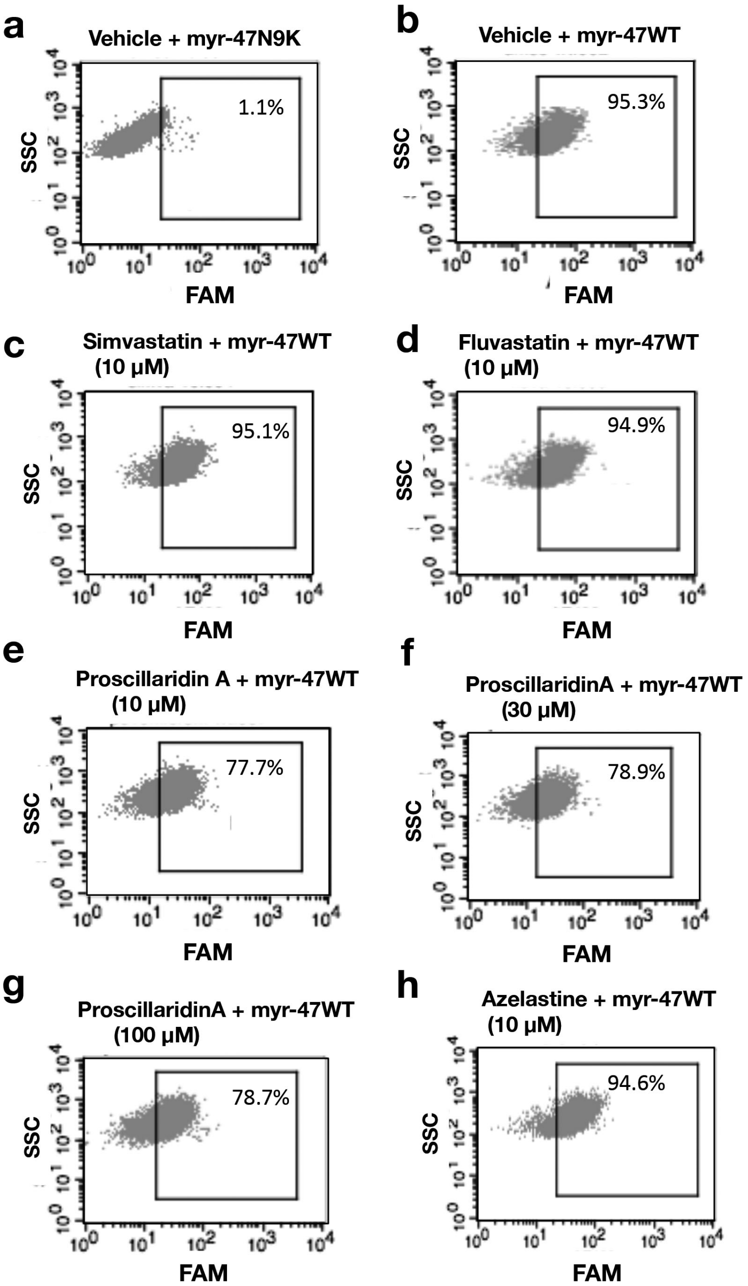

Supplementary Figure 5. Effects of compounds on the binding ability of preS1 lipopeptide to HepG2/NTCPA3 cells. a and b. HepG2/NTCPA3 cells were pretreated with DMSO for 30 min and then were treated with myr-47N9K (a) or myr-47WT (b) in the presence of DMSO for an additional 30 min. The resulting cells were subjected to FACS analysis. **c to h.** HepG2/NTCPA3 cells were pretreated with the indicated concentration of each compound for 30 min and then were treated with myr-47WT in the presence of each compound for an additional 30 min. The effects of simvastatin (c), fluvastatin (d), proscillaridin A (e, f and g), and azelastine (h) on preS1 binding were analyzed using FACS analysis, as described in the Materials and Methods section.

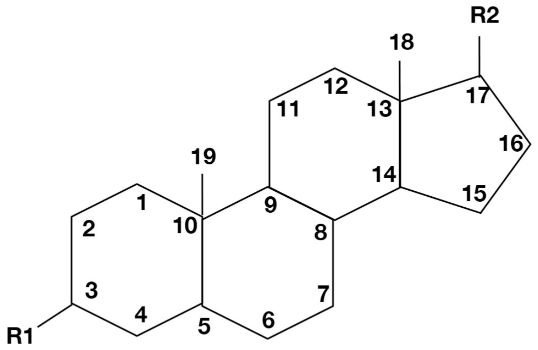

	Position					
**Compounds**	**R1**	**5**	**12**	**14**	**R2**	**19**
**Proscillaridin A**	**L-rhamnose**	**4-5 double bond**		**OH**	**δ-lactone**	
**Bufalin**	**OH**			**OH**	**δ-lactone**	
**Convallatoxin**	**L-rhamnose**	**OH**		**OH**	**γ-lactone**	**O**
**Digitoxin**	**tri-D-digitoxose**			**OH**	**γ-lactone**	
**Digoxin**	**tri-D-digitoxose**		**OH**	**OH**	**γ-lactone**	

Supplementary Figure 6. Chemical structures of digitalis-like drugs used in this study. Chemical structures of digitalis-like drugs used in this study are summarized on the basis of the report by Gozalpour et al. [1]. The basic structure of steroid ring is shown at the top, while the characteristics of each compound are described at the bottom.

Supplementary Reference

1. Gozalpour, E. et al. Interaction of digitalis-like compounds with liver uptake transporters NTCP, OATP1B1, and OATP1B3. *Mol. Pharm.* **11**, 1844-55 (2014).
